# Expression of Cyclin-D1 in Astrocytes Varies During Aging

**DOI:** 10.3389/fnagi.2018.00104

**Published:** 2018-04-24

**Authors:** Brigitte Ciapa, Sylvie Granon

**Affiliations:** CNRS, Team Neurobiology of Decision Making, Institute of Neuroscience Paris-Saclay, UMR 9197, Université Paris-Sud, Orsay, France

**Keywords:** astrocytes, cyclin-D1, GFAP, neurogenesis, cortex, hippocampus, SVZ

## Abstract

D-Cyclins control progression through the G1 phase and the G1/S transition of the cell cycle. In the adult brain, they regulate neurogenesis which is limited to the sub-granular zone of the dentate gyrus (DG) and to the sub-ventricular zone (SVZ) of the lateral ventricles. Yet, D-cyclins have also been detected in other parts of the adult brain in differentiated neurons that do not proliferate and rather die by apoptosis in response to cell cycle reactivation. Expression of D-cyclins in astrocytes has also been reported but published results, such as those concerning neurons, appear conflictual. We carried out this study in order to clarify the general pattern of D-cyclin expression in the mouse brain. By performing GFAP/cyclin-D1 double labeling experiments, we detected hypertrophic astrocytes expressing cyclin-D1 in their cytoplasmic processes. Their number increased with age in the hippocampus area but decreased with age in the SVZ. Clusters of astrocytes expressing cyclin-D1 were also detected in the cortical areas of old mice and around blood vessels of neurogenic areas. Other non-asteroidal small cells, probably stem cells, expressed both GFAP and nuclear cyclin-D1 in the neurogenic area of the DG and in the SVZ at a higher density in young mice than in old mice. Finally, cells expressing cyclin-D1 but not GFAP were also found scattered in the striatum and the CA1 region of the hippocampus, and at a high percentage in cortical layers of young and old mice. Our results suggest that astrocytes may control neuronal functions and proliferation by modulating, in normal or altered conditions such as aging or degenerative diseases, cyclin-D1 expression.

## Highlights

• GFAP/cyclin-D1 positive cells are observed by double-labeling experiments.
• Hypertrophic cyclin-D1 positive astrocytes form clusters in cortices of old mice.
• Their number increases with age in the hippocampus area but decreases with age in the SVZ.
• They are found in vascular zones close to these neurogenic areas.
• Astrocytes may control neuronal functions and neurogenesis by expressing cyclin-D1.

## Introduction

D-Cyclins belong to the family of cyclin-dependent kinases (CDKs)/cyclin complexes which regulate the cell cycle in all eukaryotes (Malumbres and Barbacid, 2009; Tyson and Novák, 2015). They play a crucial role during the G1 phase and control the G1/S transition. Three D-type cyclins have been identified, cyclin-D1, D2 and D3, that specifically activate CDK4 or CDK6, thus giving rise to distinct cyclin D-CDK4/6 complexes (Choi and Anders, 2014). Cyclin-D1 and CDK4/6 regulatory proteins show some of the highest frequencies of amplification and deletion among genes involved in human tumors (Choi and Anders, 2014).

In the brain, the CDK/cyclin system thus regulates proliferation and differentiation of neuronal precursors (neurogenesis) that give rise to new neurons and glia (Frade and Ovejero-Benito, 2015). In the adult, this process is limited to the sub-granular zone (SGZ) of the dentate gyrus (DG) (Lieberwirth et al., 2016) and to the sub-ventricular zone (SVZ) of the lateral ventricles and rostral migratory stream (Lim and Alvarez-Buylla, 2016). Low levels of neurogenesis have nevertheless been reported in other brain regions such as the neocortex and striatum (discussed in Luzzati et al., 2014). However, most differentiated neurons in the adult brain are post-mitotic cells that can indeed actively re-enter the cell cycle but only during different neurodegenerative diseases. As discussed thoroughly by Frade and Ovejero-Benito (2015), it is still not fully understood why some of these “activated neurons” replicate their DNA and survive as tetraploid neurons while others die by apoptosis as soon as they reach the S-phase. Nevertheless, CDK/cyclin complexes can regulate neuronal functions such as migration, axonal growth, dendrite formation, and synaptic plasticity (Frank and Tsai, 2009). These cell-cycle independent functions of Cdks in brain are mostly related to Cdk5 kinase, which is an unconventional member of the Cdk-family (reviewed by Su and Tsai, 2011; Lim and Kaldis, 2013). Their activity changes when neuronal functions are modified, during stress, normal training, or neurological disorders (Kawauchi et al., 2013; Frade and Ovejero-Benito, 2015; Lieberwirth et al., 2016). Therefore, differentiated neurons can be found in different areas of the normal adult brain expressing a number of regulators of G1/S transition, including cyclin-D. A role in the neural aging processes and neurodegenerative diseases has also been attributed to D-cyclins. In the brain, aging is accompanied by alterations of neuronal morphology, progressive loss of function of post-mitotic neurons and decline of neurogenesis. Neural aging often triggers aberrant entry into the cell cycle (Sarlak et al., 2013; Chow and Herrup, 2015) and mediates oxidative stress responses, mitochondrial dysfunction and DNA damage in various animals, from worms and flies to mice and humans (Bishop et al., 2010). In these processes, the activity of D-cyclins-CdK4/6 must be blocked in the case of DNA damage, to allow for repair and to prevent proliferation, as demonstrated in *Drosophila* by Icreverzi et al. (2015).

The role in neurogenesis of D-cyclins has thus been comprehensively deciphered (Kowalczyk et al., 2004; Ansorg et al., 2012). Mice lacking cyclin-D1 display neurological troubles associated with cerebello-cortico-reticular defects (Sicinski et al., 1995; Lalonde and Strazielle, 2011). Proliferation of granule cell precursors and differentiation of granule and stellate interneurons in the cerebellum are altered in cyclin D2-deficient mice (Huard et al., 1999; Kowalczyk et al., 2004). Neurogenesis in the DG is completely absent in these adult mice while developmental neurogenesis still allows formation of all major cerebral structures (Kowalczyk et al., 2004; Ansorg et al., 2012). Most other studies that decipher the expression of D-cyclins have been obtained using mouse embryos and only a few studies have been conducted using adult mice and human brains, giving results that appear rather conflictual. Kowalczyk et al. (2004) report that cyclin-D2 would only be expressed in dividing cells derived from neuronal precursors in the hippocampus, but other authors also describe the expression of cyclin-D1 as follows: in adult P60 mice, cyclin-D1 expression would be nuclear not only in proliferating astroglia of the SGZ but also in post-mitotic neurons of the CA1 hippocampal field and in pyramidal neurons in layers 3 and 5 of the cortex (Glickstein et al., 2007). A similar nuclear expression of cyclin-D1 in neurons of adult mice has been reported by Koeller et al. (2008), but these authors also found a cytoplasmic labeling in the largest motor neurons and in microglia of the brain stem and spinal cord. Finally, Sumrejkanchanakij et al. (2003) suggested that cyclin-D1 would be predominantly cytoplasmic in post-mitotic neurons but would enter the nucleus in proliferating progenitor cells. This fits with the cytoplasmic expression of cyclin-D1 in neurons of the cerebral cortex of adult mouse brain (De Falco et al., 2004).

Other studies have also involved astrocytes. These cells store glycogen and secrete glio-signaling molecules, including peptides such as apolipoprotein E (Zorec et al., 2016). In the SVZ, they constitute a mixed population of cells that have different neurogenic fate properties (Platel and Bordey, 2016). They are also closely associated with the blood–brain barrier (BBB) and the connected endothelial cells (ECs), in particular to control oxygen and glucose delivery to active neurons (Cheslow and Alvarez, 2016; Filosa et al., 2016). Astrocytes are then crucial spatio-temporal integrators that coordinate the neural network in all parts of the brain, which disintegrates during aging and during the progression of neurodegenerative diseases such as AD (Dallérac and Rouach, 2016). They provide an essential contribution in the formation and preservation of memory and are therefore involved in cognitive decline and in sensory and motor deficits that are associated with aging in animals (Zorec et al., 2016). The study of cyclin-D expression in astrocytes has led to various conflicting data found in literature similar to those concerning neurons as reported above. In adult mice, astrocytes of the DG or the neocortex would not express cyclin-D1 (Koeller et al., 2008). Conversely, Nobs et al. (2014) detected cyclin-D1 in astrocytes of cortices but the number of which remain unchanged in cyclin-D1-deficient mice. Over-expression of cyclin-D1, which promotes the proliferation of neural stem cells (NSCs), would induce their differentiation into astrocytes (Ma et al., 2015). This is contradicted by Bizen et al. (2014) who report that downregulation of cyclin-D1 would rather enhance astrogliogenesis of NSCs. Finally, GFAP-positive astrocytes expressing cyclin-D1 can be found close to amyloid plaques in brains of APP23 transgenic mice but are not seen in control mice (Gärtner et al., 2003).

In light of all these results, it seemed to us that the general pattern of cyclin-D1 expression in the mouse brain required clarification. In our first simple immunostaining experiments using a C57BL/6J 1 year old mouse, we detected cyclin-D1 in neurons scattered in the striatum, in the CA1 region of the hippocampus, in deep layers (V–VI) of the cortex and in a high percentage of pyramidal neurons of layers II–III of the cortex. Surprisingly, we also found clusters of asteroidal cyclin-D1 positive cells in brain cortical areas. The presumed identity of these cells as astrocytes led us to ask the following questions: do astrocytes normally express cyclin-D1 and, if this is the case and since we found these clusters in a 1 year old mouse, would it be related to aging? By performing GFAP/cyclin-D1 double labeling experiments, we confirmed the presumed identity of asteroidal cells expressing cyclin-D1 as being astrocytes in cortical areas of aged mice. However, these cells were also found in neurogenic areas and closed vascular zones such as the ventricular zone and the DG where their expression changed during aging. We suggest that astrocytes may modulate neuronal functions, in normal and/or in altered conditions such as aging or degenerative diseases, by mechanisms implying cyclin-D1 expression.

## Materials and Methods

### Animals

Male C57Bl/6J mice bred in Charles River’s facilities (Orleans, France) were used. Six Young Wild Type mice (about 2 months old, referred to as “young mice”) and 10 Adult Wild Type mice (about 1 year old, referred to as “old mice”) were used in these experiments. They were housed and treated according to the ethical standards defined by the National Center of the Scientific Research for Animal Health and cared for with strict compliance to EEC recommendations (no. 86/609). They were maintained in a standard rearing facility in collection cages of 2–4 mice and were never implicated in any behavioral experiment. Ethic protocol number is 2015_04.

### Immunodetection

In preliminary experiments, we observed that slices of brain, before any immunostaining protocol, gave red or green highly autofluorescent signals in common epifluorescence observations (**Supplementary Figure S1A**). This known artifact, which can lead to “convincing impostors” as well-described and discussed in Spitzer et al. (2011), has been frequently reported and is due to the presence of lipofuscin granules. We therefore tested several types of fixation followed by diverse immunostaining procedures.

#### Fixation Procedures

The following procedure, commonly used for electron microscopy observation, led to the best results after immunostaining. Mice were first perfused with buffer 1 (Bf1: Cacodylate 0.1 M, KCl 2.7 mM, CaCl_2_ 2 mM, NaCl 140 mM) and then with Bf2 (Bf1 containing 4% PFA, 0.05% Glutaraldehyde, and 0.15% Picric acid). Brains were dissected and incubated for 6 h at 4°C in Bf3 (Bf2 containing 5 mM Aprotitin, 1 mM Pefabloc, and 1 mM Pepstatin). Brains were finally transferred into PBS and kept at 4°C until microtome sectioning. 50 μm brain coronal slices were made in PBS at room temperature (RT). In multi-labeling experiments, series of 50–70 serial slices including the hippocampus were kept in 96-well microplates. In this manner, the entire region including the hippocampus was observed through 12–15 brain slices.

#### Immunostaining

In order to remove autofluorescence, brain slices were treated by immersion in 0.1% Sudan Black B (SBB, Merck AG Darmstad, Germany) in 70% ethanol for 20 min at room temperature in the dark. Slices were then washed three times for 10 min in PBS. This allowed a complete removal of autofluorescence and a very low red or green fluorescent signal after staining with the 2^nd^ antibodies (Abs) only following a staining protocol as described below (**Supplementary Figure S1**).

Slices were incubated for 1 h in blocking buffer (BF) made with PBS-T (PBS containing 2% Tween 20), 10% donkey serum and 5% non-fat dry milk (Cell Signaling). In simple immunostaining experiments, the first Ab (anti-GFAP or anti-cyclin-D1 Ab) was then added (1/500) and slices were left in this medium overnight. Slices were then rinsed three times with PBS-T, incubated for 1 h in BF with the 2^nd^ Ab (1/1000 in PBS), rinsed again three times with PBS-T and finally mounted on glass slides in Ibidi Mounting Medium (Biovalley) for microscope observation. Abs were as follows: anti-GFAP rabbit Ab (D1F4Q, Cell Signaling), anti-NeuN mouse Ab (MAB377C, Millipore), fluorescein (FITC) conjugated donkey anti-rabbit Ab (711-095-152, Jackson), rhodamine (TRITC) conjugated donkey anti-rabbit Ab (711-025-152, Jackson) or rhodamine (TRITC) conjugated donkey anti-mouse Ab (711-025-150, Jackson).

Our preliminary experiments were performed by using either an Ab from Abcam (ab52734, results not shown) or an anti-cyclin-D1 rabbit Ab (ABE 52, Millipore). Similar results were obtained but the latter Ab gave nevertheless the best signal and the lower background and was therefore used to conduct all experiments that are described in the text. We also used, as a control experiment, a third anti-cyclin-D1 Ab (AP2612-ev, S90, CliniSciences). Results were comparable to those obtained with the Ab from Millipore, although with a higher background and less intensity, as shown in the somatosensory cortical area, and the signal was lost when used in combination with its corresponding blocking peptide (**Supplementary Figure S2**).

In simple immunostaining experiments, we verified that FITC (green) and rhodamine (red) labeling did not give any red or green signals, respectively (not shown). For triple immunostaining experiments (cyclin-D1, GFAP, and Hoechst), the anti-GFAP rabbit Ab was first conjugated to rhodamine with a Mix-n-Stain Dye Antibody Labeling Kit (CF568, Biotum). In this case, slices were first incubated for 1 h in BF and then incubated overnight in this medium in the presence of the anti-cyclin-D1 Ab (1/500). Slices were rinsed three times with PBS-T, incubated for 1 h in BF with the FITC conjugated anti-rabbit Ab (1/1000 in PBS), rinsed three times with PBS-T, and then incubated again overnight with the red anti-GFAP rabbit Ab (1/500 in BF). Hoechst (1/5000) was added 1 h before a final rinse, performed three times with PBS-T and mounting on glass slides for microscope observation. The red modified anti-GFAP Ab gave the same results in simple immunostaining experiments as those depicted in **Figure 2B** (not shown). Finally, double immunostaining experiments were performed using the NeuN mouse Ab together with either the ABE 52 (Millipore) or the AP2612-ev (CliniSciences) anti-cyclin-D1 Ab, the latter being used in the presence or not of 0.1 mg/ml of the cyclin-D1 Ab blocking peptide (BP2612d, CliniSciences) as shown in **Supplementary Figure S2**. In this case, slices were first incubated for 1 h in BF and then incubated overnight in this medium in the presence of both Ab (1/500). Slices were rinsed three times with PBS-T, incubated for 1 h in BF with both the FITC conjugated anti-rabbit Ab and the anti-mouse rhodamine Ab (1/1000 in PBS), rinsed three times with PBS-T, and finally observed as described above.

### Observations and Imaging

Slices were observed by transmitted light or by epi-fluorescence with a Nikon Eclipse TE300 equipped with a 20x plan Fluor objective or a 40x oil immersion Nikon objective. Images were taken using a Nikon D600 and displayed using Adobe Photoshop CS4 software.

## Results

### Detection of Asteroidal Cells Expressing Cyclin-D1

**FIGURE 1:**
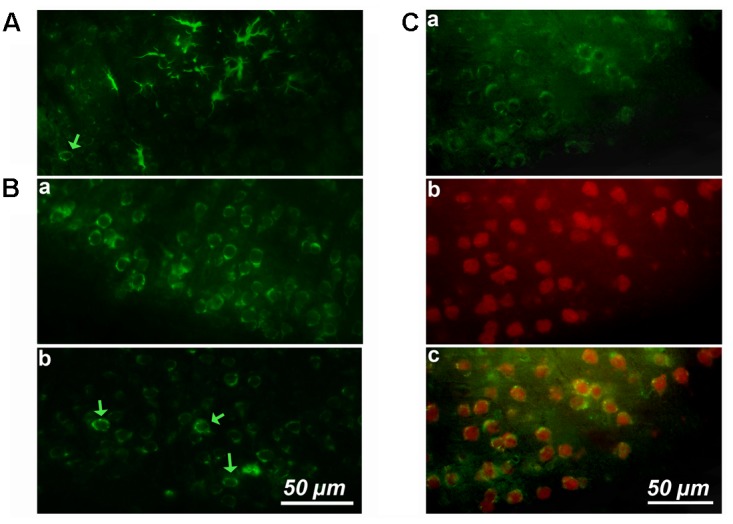
Cortical expression of cyclin-D1 in an old mouse. **(A)** Example of one cluster of asteroidal cells expressing cyclin-D1 and found in the V–VI cortical layers after simple immunostaining. One non-asteroidal cell expresses cyclin-D1 (green arrow). **(B)** Most cells expressing cyclin-D1 look like pyramidal neurons and are found at a high percentage in the layers II and III **(a)** or dispersed in deeper layers (indicated by a green arrow) **(b)**. **(C)** Images after double labeling with the anti-cyclin-D1 Ab **(a)** and the anti-Neun Ab **(b)** with the merged image **(c)**. Most cells in the layers II and III are labeled with both Abs.

**FIGURE 2:**
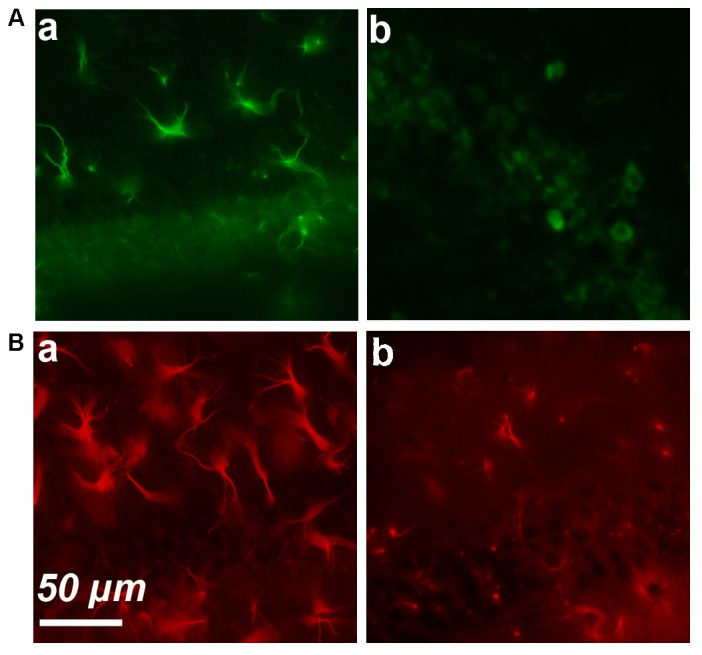
Simple immunostaining in the CA1 region in an old **(a)** and young **(b)** mouse. **(A)** Expression of cyclin-D1. **(B)** Expression of GFAP.

We started by performing simple immunostaining experiments on serial brain slices of an old mice to obtain a general image of cyclin-D1 expression. Our first observations quickly drew our attention to the presence of highly labeled asteroidal cells arranged in clusters in layers 4–5 of the somatosensory cortex (**Figure 1A**). Three other slices of brain of the same mouse were also each found to contain such clusters in layers 2–3 of the cortex. Similar observations were made in two other old mice. The same experiment was then performed on brains of younger mice, but no labeled asteroidal cells were seen in these mice. Most cells, and therefore non-asteroidal cells, in layers 2–3 of the somatosensory cortex of both young (**Figure 1Ba** and **Supplementary Figure S3**) and old mice (not shown) contained a cytoplasmic cyclin-D1 labeling. Deeper layers 4–5 contained sparse fluorescent cells (indicated by green arrows), in old (**Figure 1A**) and young mice (**Figure 1Bb** and **Supplementary Figure S3**). Most of the cells of layers 2–3 expressing cyclin-D1 were indeed neurons since they were also labeled with an anti-Neun Ab (**Figure 1C**). In fact, the highly cyclin-D1 labeled asteroidal cells were also found in the DG area of old mice (**Figure 2Aa**), while the same area in young mice only contained sparse round labeled cells located in the SGZ of the DG (**Figure 2Ab**).

These results strongly suggested that an age-dependent expression of cyclin-D1 occurred in astrocytes and led us to perform GFAP/cyclin-D1 double labeling experiments. Simple immunostaining of the hippocampus region showed high GFAP expression and hypertrophic astrocytes in old mice (**Figure 2Ba**) but a lower intensity of labeling and far less hypertrophic astrocytes in young mice (**Figure 2Bb**).

The scanning of entire brain slices from the brain part containing the hippocampus (coronal slices referred as images 63–91 in the Alan Brain Atlas) and labeled by triple immunostaining protocol (cyclin-D1, GFAP, and Hoechst) showed that GFAP staining was found throughout the brain. However, GFAP/Cyclin-D1 positive cells (indicated by white stars in all figures) were found only in the CA1, the DG, the ventricular zone, vascular areas close to these neurogenic zones and in cortices. All other astrocytes expressing GFAP only are indicated by red stars in all figures. D1-Positive cells that did not express GFAP, most likely neurons, were also found in these regions and principally in the cortex (indicated by green arrows in all figures). All these results are depicted in the details below. A few cells expressing cyclin-D1 only were also detected in other areas, although rarely, for example in the amygdala and the lateral hypothalamic area (not shown here).

### The Cortex of Old Mice Contains Clusters of Astrocytes Expressing Cyclin-D1

**FIGURE 3:**
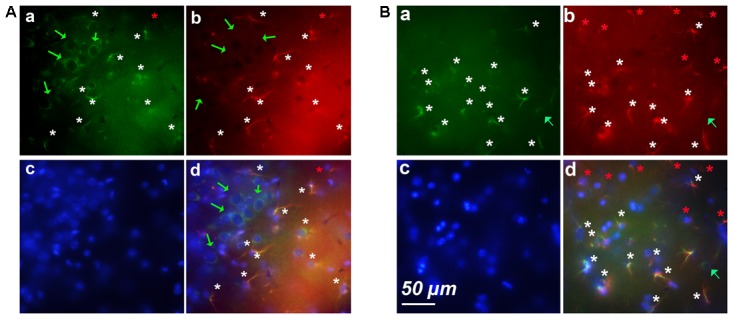
Examples of clusters of astrocytes expressing cyclin-D1 found in cortical areas of old mice. Images after triple staining performed with the anti-cyclin-D1 Ab **(a)**, the anti-GFAP Ab **(b)** and Hoechst **(c)** with the merged image **(d)**. **(A)** Layers 1–2. **(B)** Layers 5–6. Hypertrophic astrocytes labeled with both Abs are indicated by white stars, those expressing GFAP only and not cyclin-D1 by red stars, and round pyramidal cells expressing cyclin-D1 only, by green arrows.

As shown in **Figure 1B**, most neurons of layers 2–3 expressed cyclin-D only (**Figure 3Aa**). Astrocytes expressing GFAP and cyclin-D1 were found in this layer but also at the surface, in layer 1 (**Figure 3Aa**). As mentioned above, deeper layers (layers 5–6) showed sparse cells expressing cyclin-D only but also clusters of astrocytes expressing GFAP and cyclin-D1 (**Figure 3B**). In three different old mice, a total of 3, 5, and 6 clusters of 7–18 astrocytes highly labeled with the anti-cyclin-D1 Ab were seen in cortical areas per entire series of labeled slices. Such clusters of astrocytes expressing cyclin-D1 were not seen in cortices of young mice, although such astrocytes, sparse, were sometimes found in layer 1 (not shown).

### An Age-Dependent Cyclin-D1 Expression Occurs in Hypertrophic Astrocytes of CA1 and DG

**FIGURE 4:**
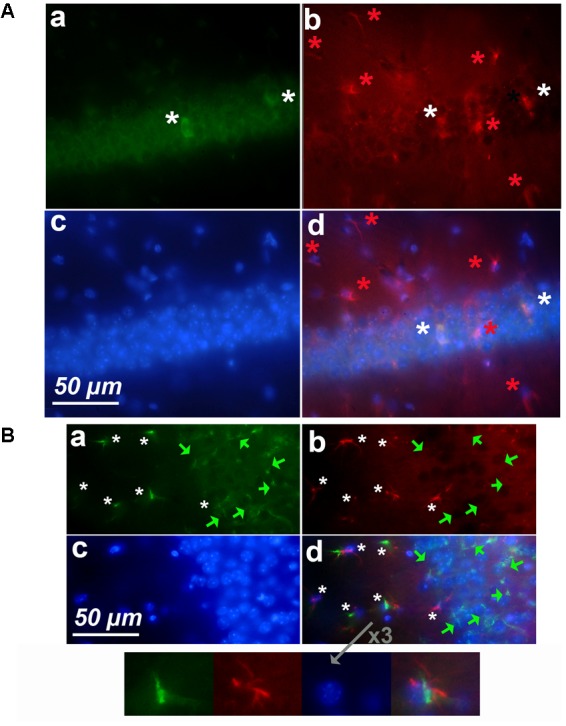
Cyclin-D1 expression in the CA1 region. Images are depicted as in **Figure 3**. **(A)** Young mouse. Rare small cells are labeled with both Abs (white stars). **(B)** Old mouse. Numerous cells are labeled with the anti- cyclin-D1 Ab only (green stars) while many hypertrophic astrocytes express both GFAP and cyclin-D1 (white stars). A 3x enlargement of one double-labeled astrocyte is shown.

In young mice, the CA1 contains sparse cyclin-D1 labeled cells that appeared small and round (**Figure 4Aa**) and GFAP positive cells, but only a few of which looked like hypertrophic astrocytes (**Figure 4Ab**). The cyclin-D1 positive small cells also express GFAP (**Figure 4Ad**). The CA1 of old mice display very different images (**Figure 4B**). A few cells expressed cyclin-D1 (**Figure 4Ba**) but not GFAP (**Figure 4Bb**) and all cells that are double-labeled with cyclin-D1 and GFAP were clearly hypertrophic astrocytes (**Figure 4Bd**). 4–5 of these astrocytes/100 μm^2^ could be counted in some parts of this area of the brain.

**FIGURE 5:**
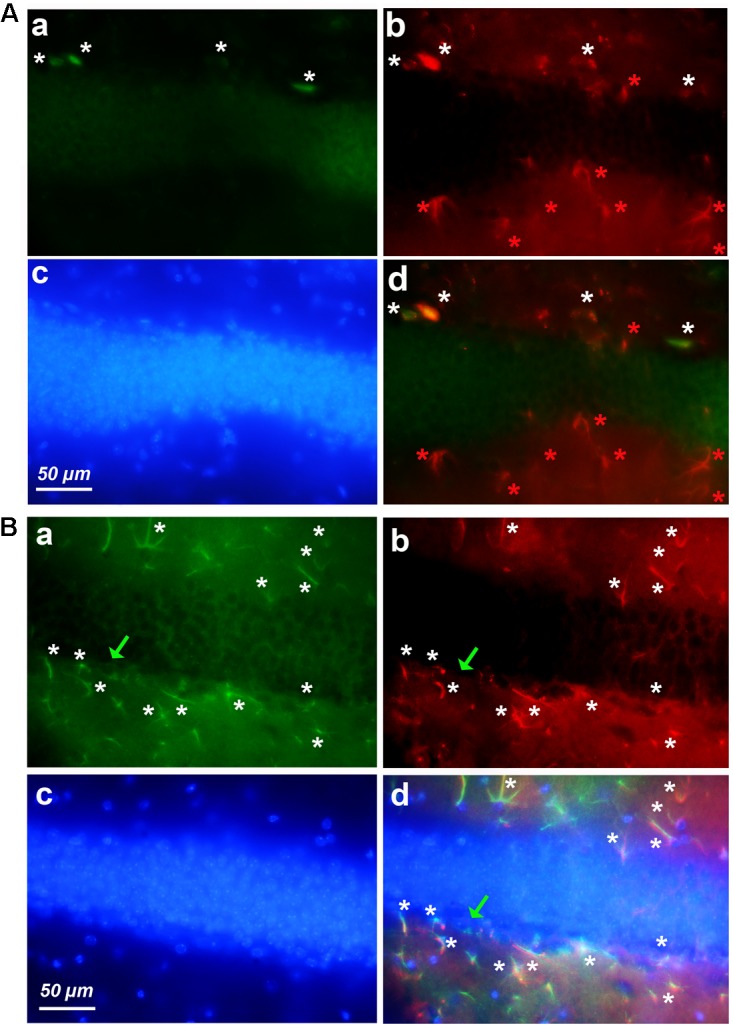
Expression of cyclin-D1 in the DG area. Staining, graphic, representation and expression in **a–c** are as explained in previous figures, the **(a,b)** images are merged in **(A)** and the **(a–c)** images are merged in **(B)**. **(A)** Young mouse. Rare small cells express both GFAP and cyclin-D1 (white stars) and all hypertrophic astrocytes express GFAP only and not cyclin-D1 (red stars). **(B)** Old mouse. Rare round cells labeled with the cyclin-D1 only can be seen (green arrow) while numerous hypertrophic astrocytes express both GFAP and cyclin-D1 (white stars).

Very similar results were obtained in the DG of young (**Figure 5A**) and old (**Figure 5B**) mice after labeling with anti-cyclin-D1 Ab (all panels a), anti-GFAP Ab (all panels b), Hoechst (all panels c), and image merging (all panels d). In young mice, a few small cells only expressed both cyclin-D1 and GFAP and were detected along the SGZ of the DG (**Figure 5Ad**). All other GFAP positive cells did not express cyclin-D1, even those that resembled hypertrophic astrocytes (**Figure 5Ad**). In old mice, very few cells expressing cyclin-D1 without any obvious GFAP were detected alongside the SGZ whereas parts of these zones in the old mouse contained more than four double labeled hypertrophic astrocytes/100 μm^2^ (**Figure 5Bd**).

### Cyclin-D1 Expression in GFAP Positive Cells of the SVZ Decreases With Aging

**FIGURE 6:**
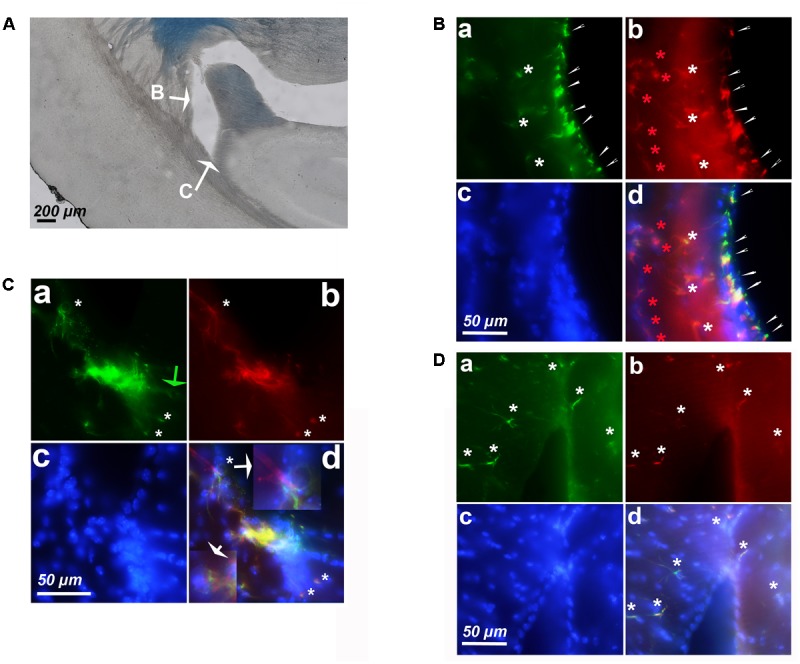
Expression of cyclin-D1 in the SVZ area in a young mouse **(A–C)** and an old mouse **(D)**. Staining and symbols in **a–d** are as explained in the previous figures. **(A)** Transmitted light image of the lateral ventricle area where fluorescent images shown in **(B,C)** have been taken. **(B)** View of the lateral edge of the ventricle. Small cells highly labeled with both anti-cyclin-D1 and anti- GFAP Abs are aligned along the edge of the ventricle (small white arrows), hypertrophic astrocytes express cyclin-D1 and GFAP behind (white stars) and those located further back express GFAP only (red stars). **(C,D)** View of the top of the ventricle near the ganglionic eminence. In the young mouse **(C)**, some cells express cyclin-D1 only with no obvious GFAP (green arrow), highly fluorescent compacted cells express both cyclin-D1 and GFAP, and a few hypertrophic astrocytes expressing both GFAP cyclin-D1 are seen (white arrows), a 2.5x enlargement of two of them is shown in **(d)**. On the contrary, a few scattered hypertrophic astrocytes expressing both cyclin-D1 and GFAP (white stars) are visible in the old mouse **(D)**.

Since we detected astrocytes expressing cyclin-D1 in the SGZ of the DG, which is a neurogenic zone, it was not surprising to find these cells in the other neurogenic zone, namely the SVZ (**Figure 6A**). In young mice, small cells highly labeled with the anti-cyclin-D1 and anti- GFAP Abs were found aligned along the lateral edges of the lateral ventricle (**Figure 6B**). Hypertrophic astrocytes, located behind these small cells, expressed cyclin-D1 and those situated further back expressed GFAP only (**Figure 6Ba,b,d**). At the top of the ventricle, near the ganglionic eminence, a mass of highly fluorescent cells expressing both cyclin-D1 and GFAP can be seen with nearby cells that express cyclin-D1only (**Figure 6C**). These zones were not as heavily fluorescent in old mice, and only a few hypertrophic astrocytes expressing both GFAP and cyclin-D1 were detected (**Figure 6D**).

### Hypertrophic Astrocytes Adjacent to Blood Vessels Express Cyclin-D1

**FIGURE 7:**
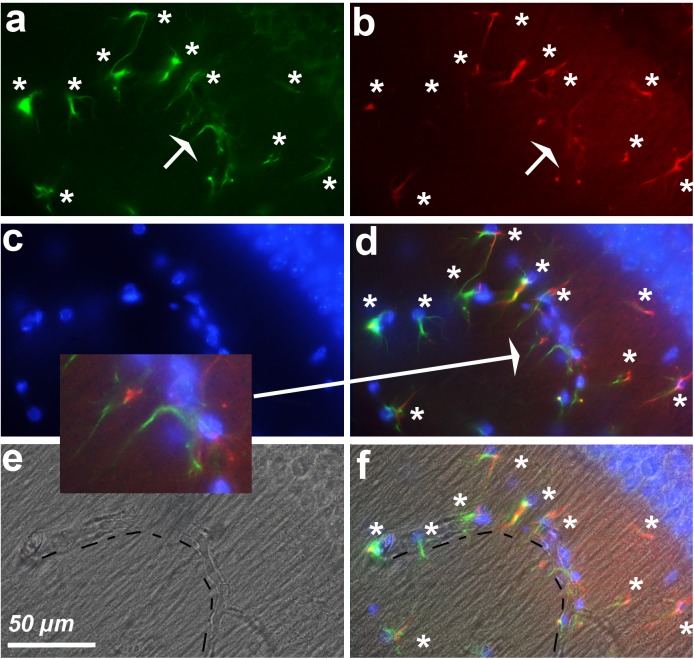
Astrocytes express cyclin-D1 along blood vessels. An area in the vicinity of the hippocampus of a young mouse is shown. **(a–d**) Shows staining and symbols as explained in the preceding figures. The shape of the blood vessel (hashed line) is indicated in the transmitted light image **(e)** and this image with that of **(d)** is merged in **(f)**. The expression of GFAP in astrocytes (white stars) appears more or less fragmented; these cells express cyclin-D1 in their long cytoplasmic processes which do not seem to contain any obvious GFAP as indicated in the 2x enlargement of **(d)**.

**FIGURE 8:**
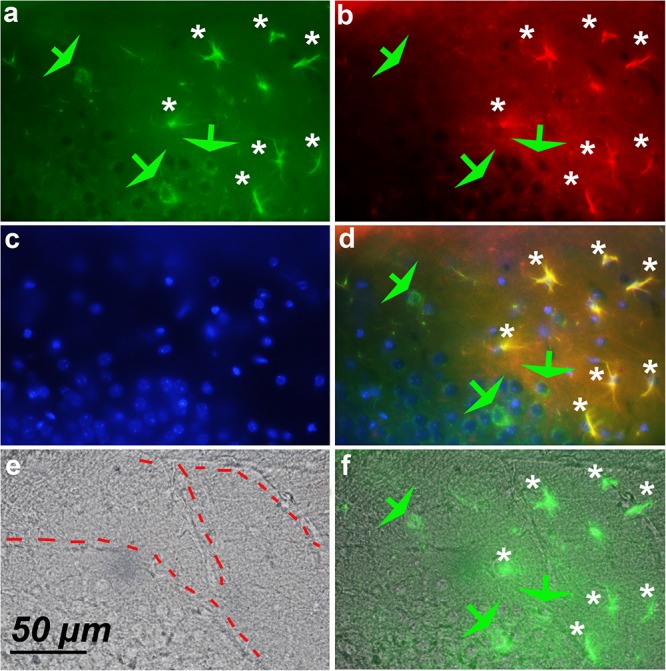
Expression of cyclin-D1 in the striatum area of a young mouse. **(a–d)** Are as explained in this figure, the merged image of **(a,e)** is shown in **(f)**. Astrocytes expressing both GFAP and cyclin-D1 are found close to the blood vessel (white stars) where some scattered neurons expressing cyclin-D1 only are also seen (green arrow).

Screening of the whole triple immunostained slices allowed us to detect areas containing 12–18 hypertrophic astrocytes expressing both GFAP and cyclin-D1 that were arranged along blood vessels in the vicinity of the hippocampus of young mice (**Figure 7**). Similar arrangements were noticed in the striatum area but, in this case, they were accompanied by a few large cells expressing cyclin-D1 only nearby (**Figure 8**). Similar images were found in old mice, except that the GFAP labeling of hypertrophic astrocytes appeared more intense as consistently observed in old mice and as displayed above (not shown).

## Discussion

Age-dependent expression of cyclin-D1 occurring in various brain areas in cells, whether or not they are astrocytes, suggest multiple roles of cyclin-D1 in the adult brain.

### Visualization of GFAP and Cyclin-D1 Expression

The first obvious question concerns the validity of our labeling experiments. We would like to point out that we did not find a single brain, out of dozens, that gave slices that were not highly autofluorescent in all protocols of fixation tested. As discussed by Spitzer et al. (2011), we believe that results showing immunofluorescence staining on slices of brain that were not pretreated with black Sudan should be considered with caution.

We used an anti-GFAP Ab to detect this protein which is the hallmark intermediate filament protein in astrocytes. Changes in the expression of this protein have been well-deciphered and correlated with the extended and thickened astrocytic processes occurring during aging, after an acute CNS trauma or ischemia, or during neurodegenerative diseases (Hol and Pekny, 2015; Yang and Wang, 2015). Our images indeed show a more fragmented GFAP staining with far less hypertrophic astrocytes in young mice compared to older mice. This type of GFAP staining, with differences noted during aging, looks like that obtained by Hayakawa et al. (2007) or Jinno (2011). However, our images in young mice show astrocytes appearing even less hypertrophic than those described by other authors such as Rodríguez et al. (2014). This is most probably due to the fact that these authors show superimposition of several confocal microscopy images, which we did not do here.

We are confident that detection of astrocytes that are co-stained with both GFAP/cyclin-D1 is not artifactitious. Firstly, cyclin-D1/FITC does not give any red light, and neither does GFAP-rhodamine emit any green light. Secondly, cells expressing cyclin-D1 but not GFAP (see for example **Figures 2A**, **3B**, **7b**) or GFAP only (**Figures 2B**, **3A**, **4A**, **6B**) are detected in double labeling experiments, ruling out the possibility of cross-immunoreactivity between these two Abs. This is also unlikely because of the very low homology between the sequence of the mouse GFAP protein and that of the human cyclin-D1 used as immunogen of the anti-cyclin-D1 Ab. Thirdly, different parts of a same cell that are not similarly labeled are also detected. This is particularly well-observed in astrocytes located around the blood vessel displayed in **Figure 7a**. Finally, three different anti-cyclin-D1 antibodies gave similar results (see section “Materials and Methods”), signals obtained with one of them being lost after incubation with the corresponding immunogenic peptide (**Supplementary Figure S2**). Signals that are more or less intense can actually be obtained using these three Abs, but this does not explain the discrepancies in intracellular localization of staining (nuclear vs. cytoplasmic staining of cortical neurons for example) reported in the literature.

### Different Types of Cells Express Cyclin-D1

We detected cells expressing cyclin-D1 but not GFAP in various areas of the brain. Firstly, a few of these cells were seen in the striatum, which corroborates a recent observation that neurogenesis is activated in lateral striatum of juvenile animals (Luzzati et al., 2014). Secondly, the CA1 region of the hippocampus of old mice contains scattered neurons expressing cyclin-D1 only, as also observed by these same authors and by Koeller et al. (2008), although these authors detected them in young mice, which we did not. As discussed by these authors, it is unlikely that all these cells are either proliferating or dying cells, and the expression of cyclin-D1 is most probably involved in the control of hippocampal circuits (Baumann and Mattingley, 2014). Thirdly, a high percentage of pyramidal neurons of layers II–III and a few scattered cells in deeper layers (V–VI) of the cortex were immunolabeled with cyclin-D1, similarly to observations made by Glickstein et al. (2007). However, these authors obtained nuclear staining while our images show an obvious cytoplasmic staining of these cortical neurons, our results being thereby more aligned to those reported by Sumrejkanchanakij et al. (2003) and De Falco et al. (2004). Again, it is more probable that expression of cyclin-D1 in these cells is related to regular neural processes rather than to proliferation or pathogenicity (Frank and Tsai, 2009; Kawauchi et al., 2013).

### Age-Dependent Expression of Cyclin-D1 Occurs in Astrocytes

Since cyclin-D1 expression of GFAP positive cells is found in areas that are either neurogenic (IC, SVZ) or not (cortex, blood vessels) and varies with age, we can hypothesize pleiotropic roles of cyclin-D1 in these cells.

Small cells, found in the neurogenic area of the DG and at a density higher in young mice than in old mice, express both GFAP and nuclear cyclin-D1. In this case, cyclin-D1 expression is most probably linked to progression through the cell cycle (Frade and Ovejero-Benito, 2015). This confirms the results of Glickstein et al. (2007) who suggest, after BrdU pulse chase experiments, that such cells would be proliferating astroglia. GFAP-expressing stem cells of the DG have been well-described (Yamaguchi et al., 2016). A very high density of these cells is also seen in the SVZ, in young mice but not in old mice. This fits with the decrease in neurogenesis that occurs during adult life and is boosted in older brains (Rusznák et al., 2016). The small GFAP/cyclin-D1 positive cells along the ventricle would be the type B1 astrocyte-like cells. Those still expressing both proteins and located backward would be the type B2 cells, the number of hypertrophic astrocytes expressing GFAP but not cyclin-D1 increasing as the depth of location (Platel and Bordey, 2016).

Besides these small cells, all GFAP/cyclin-D1 positive cells look like hypertophic astrocytes that express cyclin-D1 in their long cytoplasmic processes. The fact that GFAP expression increases with hypertrophy during aging corroborates the data of Rodríguez-Arellano et al. (2016). The astroglial adaptive plasticity as discussed by these authors would also be correlated with cyclin-D1 expression. This idea of a link between the two proteins is reinforced by the fact that over-expression of cyclin-D1 induces that of GFAP in the NSCs (Ma et al., 2015). But how could they interact between each other? GFAP and its various isoforms, as parts of the cytoskeleton of glia cells (Yang and Wang, 2015), could control migration, motility and anchoring of membrane receptors and transporters (Hol and Pekny, 2015). These highly organized cytoskeleton complex imply in particular integrins, connexins and gap junctions and allow astrocytes to make extensive contact with neurons and BBB vascular endothelial cells and pericytes, all these cells forming a very intricate network known as the neurovascular unit (Tanigami et al., 2012). As a matter of fact, expression of GFAP is bound in astrocytes to that of integrins (Moeton et al., 2014) which are part of the well-defined focal adhesions linked to the actin cytoskeleton and to a variety of other proteins such as paxillin, talin and vinculin, all expressed in astroglia (Kálmán and Szabó, 2001; Caltagarone et al., 2010). Cytoplasmic cyclin-D1 would therefore play a role in astrocytes similar to that reported in fibroblasts where it regulates cell invasion and metastasis through the phosphorylation of paxillin (Fusté et al., 2016) or to that of keratinocytes where it regulates cell–matrix adhesion (Fernández-Hernández et al., 2013). Finally, the number of hypertrophic astrocytes expressing cyclin-D1 increases with age in the hippocampus area but on the contrary decreases with age in the ventricular zone. It would therefore be interesting to investigate why some astrocytes express cyclin-D1 while others don’t and why they do so in special areas only. These results strongly suggest different roles of cyclin-D1 in hypertrophic astrocytes, being linked to the control of neuronal circuits in the hippocampus and related to neurogenesis in the ventricular zone.

It seems therefore not surprising to find astrocytes expressing cyclin-D1 located near blood vessels as discussed above (Tanigami et al., 2012). However, such clusters were sparse and found in a few areas only, in neurogenic areas such as the hippocampus zone, the vicinity of the lateral ventricle and the striatum, and never everywhere along the blood vessels. We suggest that astrocytes would express cyclin-D1 at and during defined times in response to a variety of stimuli (Fareri and Tottenham, 2016) in tiny areas as proposed by Fields et al. (2015). A short half-life of these “vascular clusters” of cyclin-D1 astrocytes would explain why they are difficult to detect, particularly in the amygdala where three images of this type were nevertheless observed among all of our experiments (not shown).

The cortical clusters of astrocytes expressing cyclin-D1 in old mice were relatively rare, and can certainly be “missed” during slicing of the brain. These images recall those of astrocytes expressing cyclins D1, E, and B1 that have been found around amyloid plaques in APP mice by Gärtner et al. (2003). Old mice that have been used in the present study were healthy animals and were not expected to form amyloid plaques. It is possible that such clusters of cyclin-D1 positive astrocytes have been formed where an inflammatory event occurred. It is also possible that astrocytes, included in the neuronal connectomes described above and which would also monitor the cerebral inputs in the cortex (Fields et al., 2015), start to express cyclin-D1 as aging progresses. In conclusion, whether the appearance of astrocytes expressing cyclin-D1 in the different parts of the brain, cortex, hippocampus and SVZ, blood vessels, is beneficial or not to maintain cognitive performances during aging is a question that can be asked. Our unexpected current results could finally lead to the idea that monitoring cyclin-D1 expressing astrocytes in the mouse brain might well be taken as a marker of cerebral fitness (Pittaras et al., 2016) since neuron–astrocyte metabolic coupling appears to play a crucial role in learning and long-term memory (Tadi et al., 2015).

## Author Contributions

BC: designed the work; acquired, analyzed, and interpreted the data; drafted the work; approved the final version to be published; agrees to be accountable for all aspects of the work in ensuring that questions related to the accuracy or integrity of any part of the work are appropriately investigated and resolved. SG: made substantial contributions to the interpretation of data; revisited the work critically for important intellectual content; approved the final version to be published; agrees to be accountable for all aspects of the work in ensuring that questions related to the accuracy or integrity of any part of the work are appropriately investigated and resolved.

## Conflict of Interest Statement

The authors declare that the research was conducted in the absence of any commercial or financial relationships that could be construed as a potential conflict of interest.

## Supplementary Material

FIGURE S1 Effect of Black Sudan (BS) treatment on autofluorescence. Transmitted light images (left panels) and fluorescent images obtained with the rhodamine (red, middle panel) or FITC (green, right panel) filters of the CA3 region of the hippocampus **(A)**, the dentate gyrus **(B)**, and the somatosensory cortical area **(C)** of a young mouse are displayed. Autofluorescence emits from slices that are not treated with BS **(a)** and high signals are visualized although no staining with the FITC or rhodamine conjugated Abs was performed. Similar low level fluorescence background is obtained after BS treatment of the slice without **(b)** or after **(c)** incubation with the FITC or with the rhodamine conjugated second Ab only.

FIGURE S2 Images after double immunostaining using the cyclin-D1 Ab from CliniSciences **(a)**, the anti-Neun Ab **(b)** with the merged image **(c)** and obtained without **(A)** or in the presence of **(B)** the competing cyclin-D1 peptide. Most cells in the layers II and III of the somatosensory cortex are labeled with both Abs (white stars, **Aa**) and a diffuse and low cyclin-D1 fluorescent signal is detected in the presence of the peptide **(Ba,c)**.

FIGURE S3 Images at low magnification (10x) of the somatosensory cortex of a young mouse after immunostaining using the anti-cyclin-D1 rabbit Ab from Millipore **(a)** and Hoechst **(b)** with the merged image **(c)**. The cortical layers I to VI are labeled as reported in the Allen brain atlas.

## References

[B1] Ansorg A. Witte O. W. Urbach A. (2012). Age-dependent kinetics of dentate gyrus neurogenesis in the absence of cyclin D2. *BMC Neurosci.* 13:46. 10.1186/1471-2202-13-46 22564330 PMC3403990

[B2] Baumann O. Mattingley J. B. (2014). Dissociable roles of the hippocampus and parietal cortex in processing of coordinate and categorical spatial information. *Front. Hum. Neurosci.* 8:73. 10.3389/fnhum.2014.00073 24596551 PMC3925887

[B3] Bishop N. A. Lu T. Yankner B. A. (2010). Neural mechanisms of ageing and cognitive decline. *Nature* 464 529–535. 10.1038/nature08983 20336135 PMC2927852

[B4] Bizen N. Inoue T. Shimizu T. Tabu K. Kagawa T. Taga T. (2014). A growth-promoting signaling component cyclin-D1 in neural stem cells has antiastrogliogenic function to execute self-renewal. *Stem Cells* 32 1602–1615. 10.1002/stem.1613 24302516

[B5] Caltagarone J. Hamilton R. L. Murdoch G. Jing Z. DeFranco D. B. Bowser R. (2010). Paxillin and hydrogen peroxide-inducible clone 5 expression and distribution in control and Alzheimer disease hippocampi. *J. Neuropathol. Exp. Neurol.* 69 356–371. 10.1097/NEN.0b013e3181d53d98 20448481 PMC2869219

[B6] Cheslow L. Alvarez J. I. (2016). Glial-endothelial crosstalk regulates blood-brain barrier function. *Curr. Opin. Pharmacol.* 26 39–46. 10.1016/j.coph.2015.09.010 26480201

[B7] Choi Y. J. Anders L. (2014). Signaling through cyclin D-dependent kinases. *Oncogene* 33 1890–1903. 10.1038/onc.2013.137 23644662

[B8] Chow H. M. Herrup K. (2015). Genomic integrity and the ageing brain. *Nat. Rev. Neurosci.* 16 672–684. 10.1038/nrn4020 26462757

[B9] Dallérac G. Rouach N. (2016). Astrocytes as new targets to improve cognitive functions. *Prog. Neurobiol.* 144 48–67. 10.1016/j.pneurobio.2016.01.003 26969413

[B10] De Falco M. Fedele V. De Luca L. Penta R. Cottone G. Cavallotti I. (2004). Evaluation cyclin-D1 expression and its subcellular distribution in mouse tissues. *J. Anat.* 205 405–412. 10.1111/j.0021-8782.2004.00347.x 15575889 PMC1571359

[B11] Fareri D. S. Tottenham N. (2016). Effects of early life stress on amygdala and striatal development. *Dev. Cogn. Neurosci.* 19 233–247. 10.1016/j.dcn.2016.04.005 27174149 PMC4912892

[B12] Fernández-Hernández R. Rafel M. Fusté N. P. Aguayo R. S. Casanova J. M. Egea J. (2013). Cyclin-D1 localizes in the cytoplasm of keratinocytes during skin differentiation and regulates cell-matrix adhesion. *Cell Cycle* 12 2510–2517. 10.4161/cc.25590 23839032 PMC3841329

[B13] Fields R. D. Woo D. H. Basser P. J. (2015). Glial regulation of the neuronal connectome through local and long-distant communication. *Neuron* 86 374–386. 10.1016/j.neuron.2015.01.014 25905811 PMC4426493

[B14] Filosa J. A. Morrison H. W. Iddings J. A. Du W. Kim K. J. (2016). Beyond neurovascular coupling, role of astrocytes in the regulation of vascular tone. *Neuroscience* 323 96–109. 10.1016/j.neuroscience.2015.03.064 25843438 PMC4592693

[B15] Frade J. M. Ovejero-Benito M. C. (2015). Neuronal cell cycle: the neuron itself and its circumstances. *Cell Cycle* 14 712–720. 10.1080/15384101.2015.1004937 25590687 PMC4418291

[B16] Frank C. L. Tsai L. H. (2009). Alternative functions of core cell cycle regulators in neuronal migration, neuronal maturation, and synaptic plasticity. *Neuron* 62 312–326. 10.1016/j.neuron.2009.03.029 19447088 PMC2757047

[B17] Fusté N. P. Fernández-Hernández R. Cemeli T. Mirantes C. Pedraza N. Rafel M. (2016). Cytoplasmic cyclin-D1 regulates cell invasion and metastasis through the phosphorylation of paxillin. *Nat. Commun.* 7:11581. 10.1038/ncomms11581 27181366 PMC4873647

[B18] Gärtner U. Brückner M. K. Krug S. Schmetsdorf S. Staufenbiel M. Arendt T. (2003). Amyloid deposition in APP23 mice is associated with the expression of cyclins in astrocytes but not in neurons. *Acta Neuropathol.* 106 535–544. 10.1007/s00401-003-0760-8 12923647

[B19] Glickstein S. B. Alexander S. Ross M. E. (2007). Differences in cyclin D2 and D1 protein expression distinguish forebrain progenitor subsets. *Cereb Cortex* 17 632–642. 10.1093/cercor/bhk008 16627858

[B20] Hayakawa N. Kato H. Araki T. (2007). Age-related changes of astrocytes, oligodendrocytes and microglia in the mouse hippocampal CA1 sector. *Mech. Ageing Dev.* 128 311–316. 10.1016/j.mad.2007.01.005 17350671

[B21] Hol E. M. Pekny M. (2015). Glial fibrillary acidic protein (GFAP) and the astrocyte intermediate filament system in diseases of the central nervous system. *Curr. Opin. Cell Biol.* 32 121–130. 10.1016/j.ceb.2015.02.004 25726916

[B22] Huard J. M. Forster C. C. Carter M. L. Sicinski P. Ross M. E. (1999). Cerebellar histogenesis is disturbed in mice lacking cyclin D2. *Development* 126 1927–1935. 10101126 10.1242/dev.126.9.1927

[B23] Icreverzi A. de la Cruz A. F. Walker D. W. Edgar B. A. (2015). Changes in neuronal CycD/Cdk4 activity affect aging, neurodegeneration, and oxidative stress. *Aging Cell* 14 896–906. 10.1111/acel.12376 26219626 PMC4568977

[B24] Jinno S. (2011). Regional and laminar differences in antigen profiles and spatial distributions of astrocytes in the mouse hippocampus, with reference to aging. *Neuroscience* 180 41–52. 10.1016/j.neuroscience.2011.02.013 21320577

[B25] Kálmán M. Szabó A. (2001). Immunohistochemical investigation of actin-anchoring proteins vinculin, talin and paxillin in rat brain following lesion: a moderate reaction, confined to the astroglia of brain tracts. *Exp. Brain Res.* 139 426–434. 10.1007/s002210100789 11534866

[B26] Kawauchi T. Shikanai M. Kosodo Y. (2013). Extra-cell cycle regulatory functions of cyclin-dependent kinases (CDK) and CDK inhibitor proteins contribute to brain development and neurological disorders. *Genes Cells* 18 176–194. 10.1111/gtc.12029 23294285 PMC3594971

[B27] Koeller H. B. Ross M. E. Glickstein S. B. (2008). Cyclin-D1 in excitatory neurons of the adult brain enhances kainate-induced neurotoxicity. *Neurobiol. Dis.* 31 230–241. 10.1016/j.nbd.2008.04.010 18585919 PMC2614463

[B28] Kowalczyk A. Filipkowski R. K. Rylski M. Wilczynski G. M. Konopacki F. A. Jaworski J. (2004). The critical role of cyclin D2 in adult neurogenesis. *J. Cell Biol.* 167 209–213. 10.1083/jcb.200404181 15504908 PMC2172537

[B29] Lalonde R. Strazielle C. (2011). Brain regions and genes affecting limb-clasping responses. *Brain Res. Rev.* 67 252–259. 10.1016/j.brainresrev.2011.02.005 21356243

[B30] Lieberwirth C. Pan Y. Liu Y. Zhang Z. Wang Z. (2016). Hippocampal adult neurogenesis: its regulation and potential role in spatial learning and memory. *Brain Res.* 1644 127–140. 10.1016/j.brainres.2016.05.015 27174001 PMC5064285

[B31] Lim D. A. Alvarez-Buylla A. (2016). The adult ventricular-subventricular zone (V-SVZ) and olfactory bulb (OB) neurogenesis. *Cold Spring Harb. Perspect. Biol.* 8:a018820. 10.1101/cshperspect.a018820 27048191 PMC4852803

[B32] Lim S. Kaldis P. (2013). Cdks, cyclins and CKIs: roles beyond cell cycle regulation. *Development* 140 3079–3093. 10.1242/dev.091744 23861057

[B33] Luzzati F. Nato G. Oboti L. Vigna E. Rolando C. Armentano M. (2014). Quiescent neuronal progenitors are activated in the juvenile guinea pig lateral striatum and give rise to transient neurons. *Development* 141 4065–4075. 10.1242/dev.107987 25336736

[B34] Ma J. Cui B. Ding X. Wei J. Cui L. (2015). Over-expression cyclin-D1 promotes NSCs proliferation and induces the differentiation into astrocytes via Jak-STAT3 pathways. *Neurochem. Res.* 40 1681–1690. 10.1007/s11064-015-1635-9 26162780

[B35] Malumbres M. Barbacid M. (2009). Cell cycle, CDKs and cancer: a changing paradigm. *Nat. Rev. Cancer* 9 153–166. 10.1038/nrc2602 19238148

[B36] Moeton M. Kanski R. Stassen O. M. Sluijs J. A. Geerts D. van Tijn P. (2014). Silencing GFAP isoforms in astrocytoma cells disturbs laminin-dependent motility and cell adhesion. *FASEB J.* 28 2942–2954. 10.1096/fj.13-245837 24696300

[B37] Nobs L. Baranek C. Nestel S. Kulik A. Kapfhammer J. Nitsch C. (2014). Stage-specific requirement for cyclin-D1 in glial progenitor cells of the cerebral cortex. *Glia* 62 829–839. 10.1002/glia.22646 24550001

[B38] Pittaras E. Callebert J. Chennaoui M. Rabat A. Granon S. (2016). Individual behavioral and neurochemical markers of unadapted decision-making processes in healthy inbred mice. *Brain Struct. Funct.* 221 4615–4629. 10.1007/s00429-016-1192-2 26860089 PMC5102946

[B39] Platel J. C. Bordey A. (2016). The multifaceted subventricular zone astrocyte: from a metabolic and pro-neurogenic role to acting as a neural stem cell. *Neuroscience* 323 20–28. 10.1016/j.neuroscience.2015.10.053 26546469 PMC4821790

[B40] Rodríguez J. J. Yeh C. Y. Terzieva S. Olabarria M. Kulijewicz-Nawrot M. Verkhratsky A. (2014). Complex and region-specific changes in astroglial markers in the aging brain. *Neurobiol. Aging* 35 15–23. 10.1016/j.neurobiolaging.2013.07.002 23969179

[B41] Rodríguez-Arellano J. J. Parpura V. Zorec R. Verkhratsky A. (2016). Astrocytes in physiological aging and Alzheimer’s disease. *Neuroscience* 323 170–182. 10.1016/j.neuroscience.2015.01.007 25595973

[B42] Rusznák Z. Henskens W. Schofield E. Kim W. S. Fu Y. (2016). Adult neurogenesis and gliogenesis: possible mechanisms for neurorestoration. *Exp. Neurobiol.* 25 103–112. 10.5607/en.2016.25.3.103 27358578 PMC4923354

[B43] Sarlak G. Jenwitheesuk A. Chetsawang B. Govitrapong P. (2013). Effects of melatonin on nervous system aging: neurogenesis and neurodegeneration. *J. Pharmacol. Sci.* 123 9–24. 10.1254/jphs.13R01SR 23985544

[B44] Sicinski P. Donaher J. L. Parker S. B. Li T. Fazeli A. Gardner H. (1995). Cyclin-D1 provides a link between development and oncogenesis in the retina and breast. *Cell* 82 621–630. 10.1016/0092-8674(95)90034-9 7664341

[B45] Spitzer N. Sammons G. S. Price E. M. (2011). Autofluorescent cells in rat brain can be convincing impostors in green fluorescent reporter studies. *J. Neurosci. Methods* 197 48–55. 10.1016/j.jneumeth.2011.01.029 21310182 PMC3099411

[B46] Su S. C. Tsai L. H. (2011). Cyclin-dependent kinases in brain development and disease. *Annu. Rev. Cell Dev. Biol.* 27 465–491. 10.1146/annurev-cellbio-092910-154023 21740229

[B47] Sumrejkanchanakij P. Tamamori-Adachi M. Matsunaga Y. Eto K. Ikeda M. A. (2003). Role of cyclin-D1 cytoplasmic sequestration in the survival of postmitotic neurons. *Oncogene* 22 8723–8730. 10.1038/sj.onc.1206870 14647467

[B48] Tadi M. Allaman I. Lengacher S. Grenningloh G. Magistretti P. J. (2015). Learning-induced gene expression in the hippocampus reveals a role of neuron -astrocyte metabolic coupling in long term memory. *PLoS One* 10:e0141568. 10.1371/journal.pone.0141568 26513352 PMC4625956

[B49] Tanigami H. Okamoto T. Yasue Y. Shimaoka M. (2012). Astroglial integrins in the development and regulation of neurovascular units. *Pain Res. Treat.* 2012:964652. 10.1155/2012/964652 23304493 PMC3529429

[B50] Tyson J. J. Novák B. (2015). Models in biology: lessons from modeling regulation of the eukaryotic cell cycle. *BMC Biol.* 13:46. 10.1186/s12915-015-0158-9 26129844 PMC4486427

[B51] Yamaguchi M. Seki T. Imayoshi I. Tamamaki N. Hayashi Y. Tatebayashi Y. (2016). Neural stem cells and neuro/gliogenesis in the central nervous system: understanding the structural and functional plasticity of the developing, mature, and diseased brain. *J. Physiol. Sci.* 66 197–206. 10.1007/s12576-015-0421-4 26578509 PMC4823343

[B52] Yang Z. Wang K. K. (2015). Glial fibrillary acidic protein: from intermediate filament assembly and gliosis to neurobiomarker. *Trends Neurosci.* 38 364–374. 10.1016/j.tins.2015.04.003 25975510 PMC4559283

[B53] Zorec R. Parpura V. Vardjan N. Verkhratsky A. (2016). Astrocytic face of Alzheimer’s disease. *Behav. Brain Res.* 322 250–257. 10.1016/j.bbr.2016.05.021 27173426

