# Supplementary figures and images for: Expression of Cyclin-D1 in Astrocytes Varies During Aging
Source: Front Aging Neurosci. 2018 Apr 24;10:104. doi: 10.3389/fnagi.2018.00104 (PMC5928257; doi:10.3389/fnagi.2018.00104)

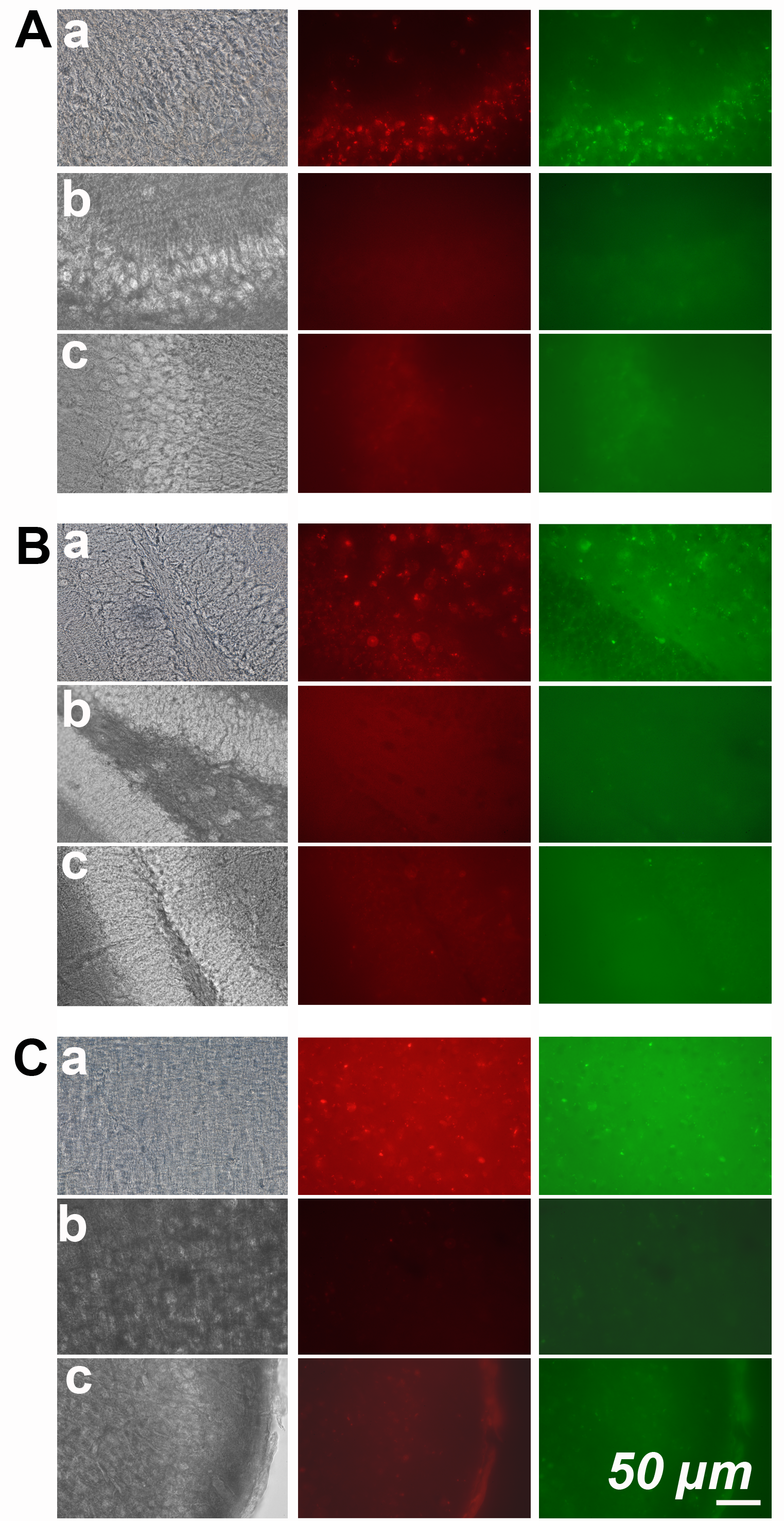

Supplement: FIGURE S1 — Effect of Black Sudan (BS) treatment on autofluorescence. Transmitted light images (left panels) and fluorescent images obtained with the rhodamine (red, middle panel) or FITC (green, right panel) filters of the CA3 region of the hippocampus (A), the dentate gyrus (B), and the somatosensory cortical area (C) of a young mouse are displayed. Autofluorescence emits from slices that are not treated with BS (a) and high signals are visualized although no staining with the FITC or rhodamine conjugated Abs was performed. Similar low level fluorescence background is obtained after BS treatment of the slice without (b) or after (c) incubation with the FITC or with the rhodamine conjugated second Ab only. [file Image_1.TIF]

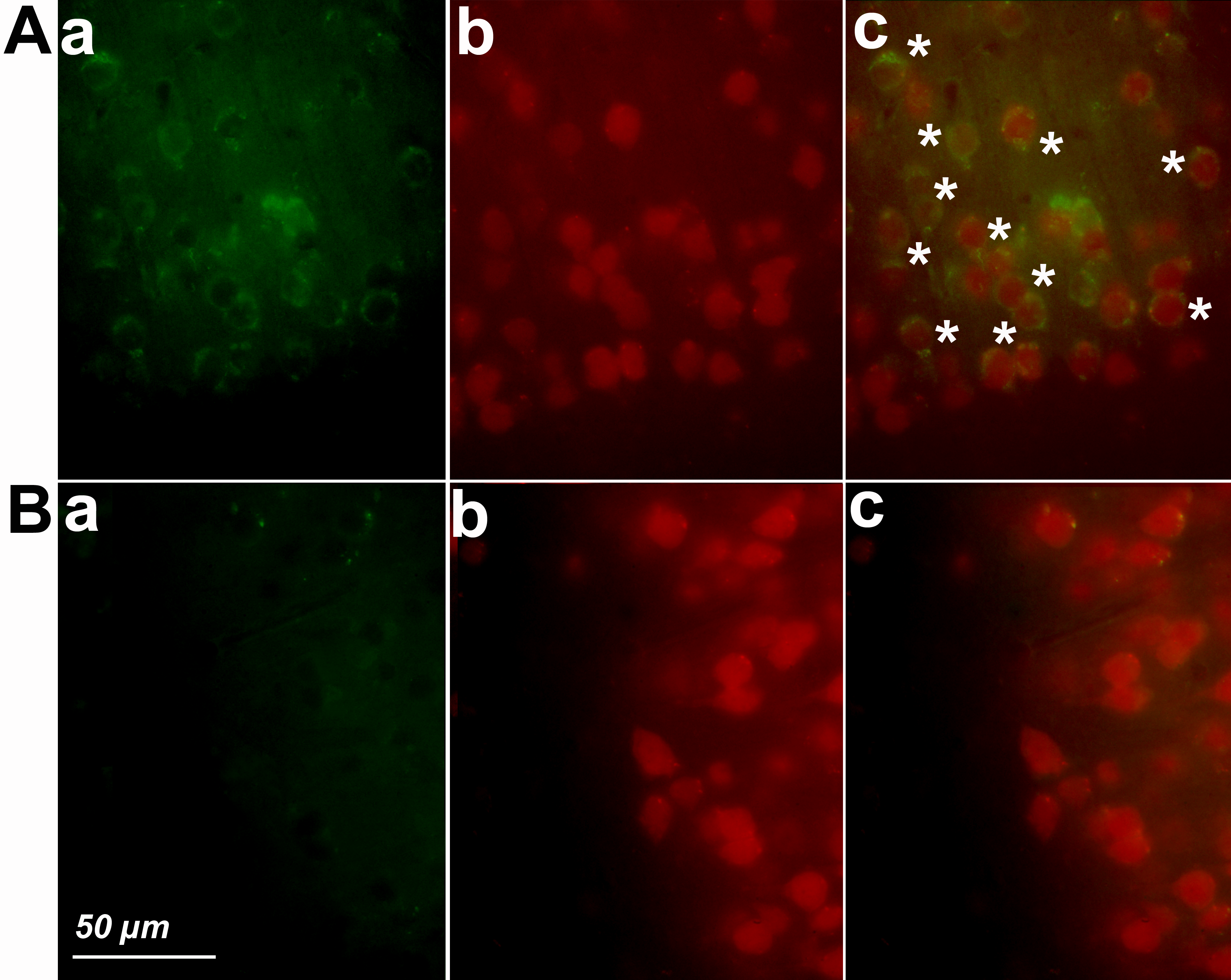

Supplement: FIGURE S2 — Images after double immunostaining using the cyclin-D1 Ab from CliniSciences (a), the anti-Neun Ab (b) with the merged image (c) and obtained without (A) or in the presence of (B) the competing cyclin-D1 peptide. Most cells in the layers II and III of the somatosensory cortex are labeled with both Abs (white stars, Aa) and a diffuse and low cyclin-D1 fluorescent signal is detected in the presence of the peptide (Ba,c). [file Image_2.TIF]

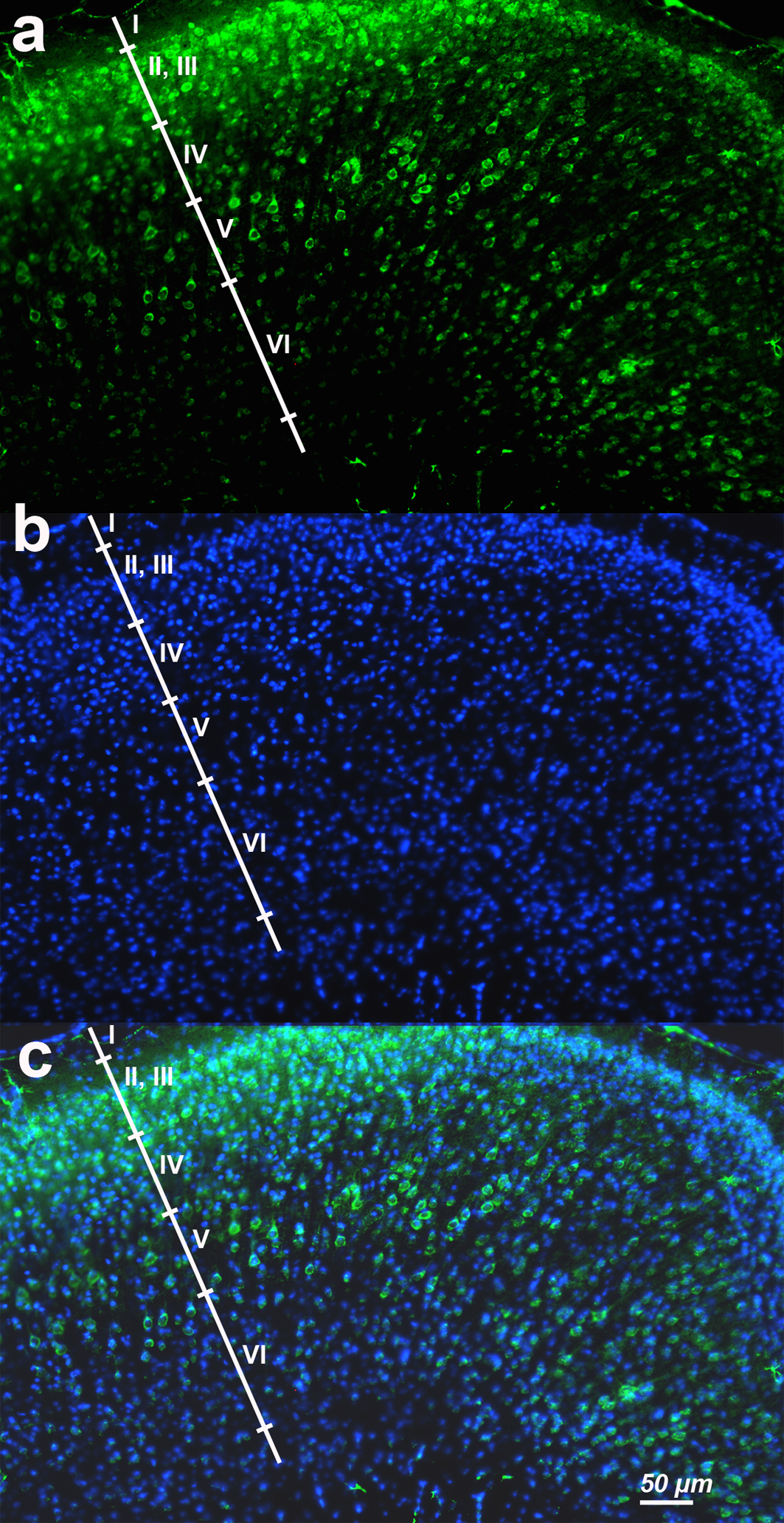

Supplement: FIGURE S3 — Images at low magnification (10x) of the somatosensory cortex of a young mouse after immunostaining using the anti-cyclin-D1 rabbit Ab from Millipore (a) and Hoechst (b) with the merged image (c). The cortical layers I to VI are labeled as reported in the Allen brain atlas. [file Image_3.TIF]
